# Antifouling Activity of Synthetic Alkylpyridinium Polymers Using the Barnacle Model

**DOI:** 10.3390/md12041959

**Published:** 2014-04-02

**Authors:** Veronica Piazza, Ivanka Dragić, Kristina Sepčić, Marco Faimali, Francesca Garaventa, Tom Turk, Sabina Berne

**Affiliations:** 1 ISMAR—CNR Institute of Marine Science, U.O.S. Genova, Via De Marini 6, 16149 Genova, Italy; E-Mails: veronica.piazza@ge.ismar.cnr.it (V.P.); marco.faimali@ismar.cnr.it (M.F.); francesca.garaventa@ismar.cnr.it (F.G.); 2 Department of Biology, Biotechnical Faculty, University of Ljubljana, Večna pot 111, Ljubljana 1000, Slovenia; E-Mails: ivana84@gmail.com (I.D.); kristina.sepcic@bf.uni-lj.si (K.S.); tom.turk@bf.uni-lj.si (T.T.)

**Keywords:** *Amphibalanus amphitrite*, antifouling, natural product antifoulants, alkylpyridinium polymers, *Haliclona (Rhizoniera) sarai*, barnacle, settlement assay, toxicity assay, swimming inhibition assay

## Abstract

Polymeric alkylpyridinium salts (poly-APS) isolated from the Mediterranean marine sponge, *Haliclona (Rhizoniera) sarai*, effectively inhibit barnacle larva settlement and natural marine biofilm formation through a non-toxic and reversible mechanism. Potential use of poly-APS-like compounds as antifouling agents led to the chemical synthesis of monomeric and oligomeric 3-alkylpyridinium analogues. However, these are less efficient in settlement assays and have greater toxicity than the natural polymers. Recently, a new chemical synthesis method enabled the production of poly-APS analogues with antibacterial, antifungal and anti-acetylcholinesterase activities. The present study examines the antifouling properties and toxicity of six of these synthetic poly-APS using the barnacle (*Amphibalanus amphitrite*) as a model (cyprids and II stage nauplii larvae) in settlement, acute and sub-acute toxicity assays. Two compounds, APS8 and APS12-3, show antifouling effects very similar to natural poly-APS, with an anti-settlement effective concentration that inhibits 50% of the cyprid population settlement (EC_50_) after 24 h of 0.32 mg/L and 0.89 mg/L, respectively. The toxicity of APS8 is negligible, while APS12-3 is three-fold more toxic (24-h LC_50_: nauplii, 11.60 mg/L; cyprids, 61.13 mg/L) than natural poly-APS. This toxicity of APS12-3 towards nauplii is, however, 60-fold and 1200-fold lower than that of the common co-biocides, Zn- and Cu-pyrithione, respectively. Additionally, exposure to APS12-3 for 24 and 48 h inhibits the naupliar swimming ability with respective IC_50_ of 4.83 and 1.86 mg/L.

## 1. Introduction

Marine biofouling is a dynamic natural process that occurs on ocean-submerged surfaces and leads to the undesired accumulation of organic polymers and of microbial, plant and animal communities and their by-products [1]. Although there is a wide diversity of fouling organisms and a variety of contributing environmental factors, a general sequence of fouling events is frequently observed [2]. The initial conditioning of the submerged surfaces by the adsorption of organic macromolecules is followed by the attachment of marine bacteria, which form a complex multi-species biofilm [3,4]. The complexity of “microfoulers” is further increased when fungi, diatoms and protozoa colonize this microbial slime layer [5]. Within hours to days, “soft macrofouling” is observed, as algal spores and various invertebrate larvae begin to attach and develop [6,7]. Shelled invertebrates, like barnacles, mussels and tubeworms, represent the “hard macrofouling” phase, which results in the formation of a mature fouling community [8]. 

The settlement and accumulation of marine organisms is a severe problem on engineered structures that are submerged in the sea, and it incurs substantial economic costs in the shipping [9,10], desalination [11] and offshore oil and gas [12] industries and in marine aquaculture [13]. Traditionally, the most effective strategy for controlling biofouling has been achieved using paints and antifouling coatings that contain toxic constituents (e.g., Cu, Zn) or biocides (e.g., tributyltin, bis(tributyltin) oxide) [14,15]. However, the accumulation of these compounds in harbors and ports led to massive pollution problems [14] and had detrimental effects on non-target marine organisms [16]. Consequently, the use of organotin-based antifouling coatings was prohibited by the Antifouling System Convention of the International Maritime Organization (effective from 17 September 2008). 

Modern antifouling approaches are investigating behavioral, chemical and physical defense mechanisms that have evolved in living organisms, to translate these into novel antifouling applications [17]. The creation of self-polishing and foul-release coatings has been inspired by the skin of marine mammals and fish, which respond to environmental stimuli, like temperature and pH [1]. Bio-inspired physical antifouling strategies have exploited the physical properties for fouling prevention, such as the surface energy and microtopography [1,15,18,19,20]. Antifoulants based on natural products have been proposed as one of the best ecologically relevant antifouling solutions [21,22], due to their lower toxicity, reversible effects at lower effective concentrations and biodegradability, compared with conventional biocides [23]. Natural antifouling compounds are produced as secondary metabolites by a wide range of organisms, and they include terpenoids, steroids, carotenoids, phenolics, furanones, alkaloids, peptides and lactones [24,25,26]. 

**Figure 1:**
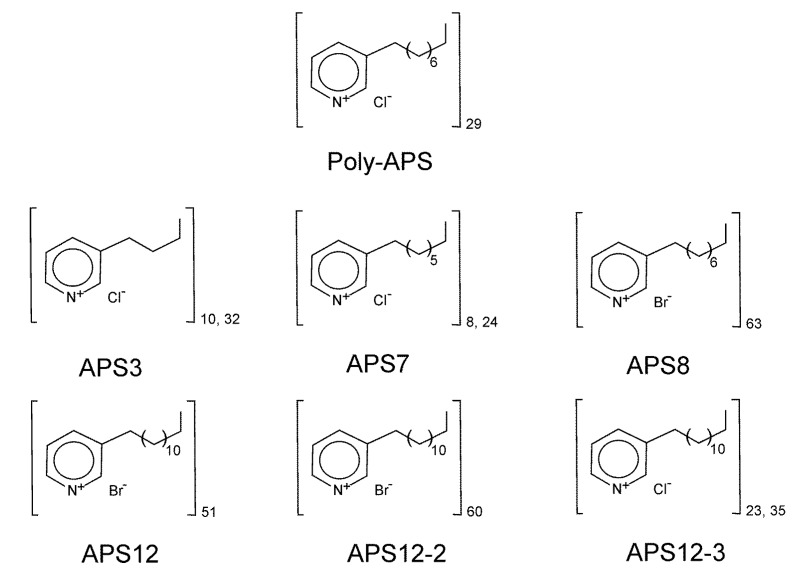
Chemical structures of natural polymeric 3-alkylpyridinium salts (poly-APS) and the synthetic poly-APS investigated. Molecular weights: natural poly-APS, 5.52 kDa; APS3, 1.46 kDa; APS7, 2.33 kDa; APS8, 11.9 kDa; APS12, 12.5 kDa; APS12-2, 14.7 kDa; APS12-3, 6.08 kDa.

To date, over 80 different bioactive 3-alkylpyridinium and 3-alkylpyridine compounds have been identified in marine sponges of the order Haplosclerida [27,28]. One of the most studied of these compounds are water-soluble polymeric 3-alkylpyridinium salts (poly-APS), secondary metabolites produced by the Mediterranean marine sponge, *Haliclona (Rhizoniera) sarai* [29]. Poly-APS (5.52 kDa; [30]) have been chemically defined as polymers that are composed of 29 monomeric *N*-butyl-3-butyl pyridinium units, with 3-octyl chains linked to the nitrogen of the adjacent unit in a head-to-tail organization (see Figure 1). In aqueous solutions, poly-APS behave as cationic detergents at concentrations >0.23 g/L and form large supramolecular structures of a 23 nm mean hydrodynamic radius [31]. At concentrations >1 g/L, poly-APS have toxic and lethal effects in rodents upon intravenous administration [23]. 

Among the numerous biological activities of poly-APS that were detailed by [27], their antifouling activity is of particular interest for the current study. In a static laboratory bioassay [2] using the most common hard macrofouler species, the barnacle, *Amphibalanus amphitrite*, poly-APS effectively deterred the settlement of cyprids (effective concentration that inhibits 50% of the cyprid population settlement (EC_50_), 0.27 mg/L), with low toxicity towards nauplii (lethal concentration that kills 50% of the cyprid population (LC_50_), 30.01 mg/L) and the non-target organisms, the alga, *Tetraselmis suecica*, and the mussel, *Mytilus galloprovincialis* [32]. Additionally, in a laboratory anti-microfouling activity assay and in the range of 0.1 mg/L to 1.0 mg/L, poly-APS prevented the formation of the natural marine biofilm, through inhibition of the growth of certain marine bacteria [33]. The molecular mechanisms behind poly-APS antifouling activity presumably involve neurotransmission blockade/modulation [34] through acetylcholinesterase inhibition, combined with their surfactant-like properties that decrease the surface tension [28]. 

Despite extensive studies of such natural antifoulants over the past 20 years, their incorporation into antifouling paints has been hampered by their limited supply [24]. Sufficient amounts of target poly-APS for broad biological screening and of poly-APS analogues for structure-activity relationship studies can be supplied through synthetic organic chemistry [35]. Using such an approach, dimers and tetramers of linear 3-alkylpyridinium salts have been produced that have high antibacterial and moderate anti-acetylcholinesterase activities [36]. In the barnacle anti-settlement assay, these oligomeric compounds did not reach the antifouling potential of natural poly-APS and were considerably more toxic [37]. In contrast, screening for novel antimicrobial agents has revealed that some of these APS analogues have considerable antibacterial activity towards biofilm-forming marine bacteria and has indicated that their charged pyridinium moiety and bromine atom, and the length of their alkyl chain, are decisive factors in this bioactivity [38]. Recently, new microwave-assisted polymerization has allowed the production of high-molecular-weight analogues of poly-APS [39,40]. Certain poly-APS analogues have been seen to behave similarly to natural poly-APS when their antifungal, antibacterial, anti-acetylcholinesterase, antitumoral and membrane-damaging activities have been assessed [39,40,41]. 

In the present study, we investigated the antifouling activity of six synthetic poly-APS analogues using *A. amphitrite* cyprids as the macrofouling model in a static laboratory settlement assay. In parallel, we examined the toxicity towards *A. amphitrite* cyprids after 24 h, 48 h and 72 h of exposure to these synthetic poly-APS and calculated their therapeutic ratios. We monitored the acute and sub-acute toxicity towards II stage *A. amphitrite* nauplii after 24 h and 48 h exposure to these poly-APS analogues. Finally, the behavior of II stage nauplii was studied for one of the most promising analogues, APS12-3, using swimming speed alteration assay as a measure of sub-lethal toxicity.

## 2. Results and Discussion

Marine biofouling is a complex and dynamic natural process that is very difficult to reproduce under laboratory conditions. As direct evaluation of antifouling coatings *in situ* is expensive and time-consuming, several bioassays have been developed to estimate the antifouling potential of novel natural products [2]. Due to the large diversity of organisms implicated in the marine biofouling process, it has been recommended that as many target species are used as possible, taken from both the microfouling and macrofouling communities [25]. Generally, bacteria, diatoms and fungi isolated from marine biofilms are studied in microfouling bioassays, while sessile hard-foulers (e.g., barnacles, tube worms, mussels) and soft-foulers (e.g., the bryozoan, *Bugula neritina*, the polychaete, *Hydroides elegans*) or seaweed (e.g., *Ulva*) are frequently used as representative macrofouling organisms. In our previous studies [32,33,42], we demonstrated that natural poly-APS can effectively inhibit the settlement and/or growth of different target fouling organisms. Using synthetic monomeric and oligomeric analogues of natural poly-APS, we also demonstrated their considerable antimicrobial activity [38]. However, in the anti-settlement assay against *A. amphitrite* cyprids, we identified only one synthetic poly-APS analogue (1,8-di(3-pyridyl)octane) that showed similar efficacy to natural poly-APS, although with a noticeably different toxic mechanism of action [37]. 

In the present study, we focused on the antifouling activities of synthetic poly-APS using *A. amphitrite* as the primary invertebrate model for biofouling [43]. The structures of the synthetic poly-APS evaluated in this study are illustrated in Figure 1.

### 2.1. Anti-Settlement Assay

**Figure 2:**
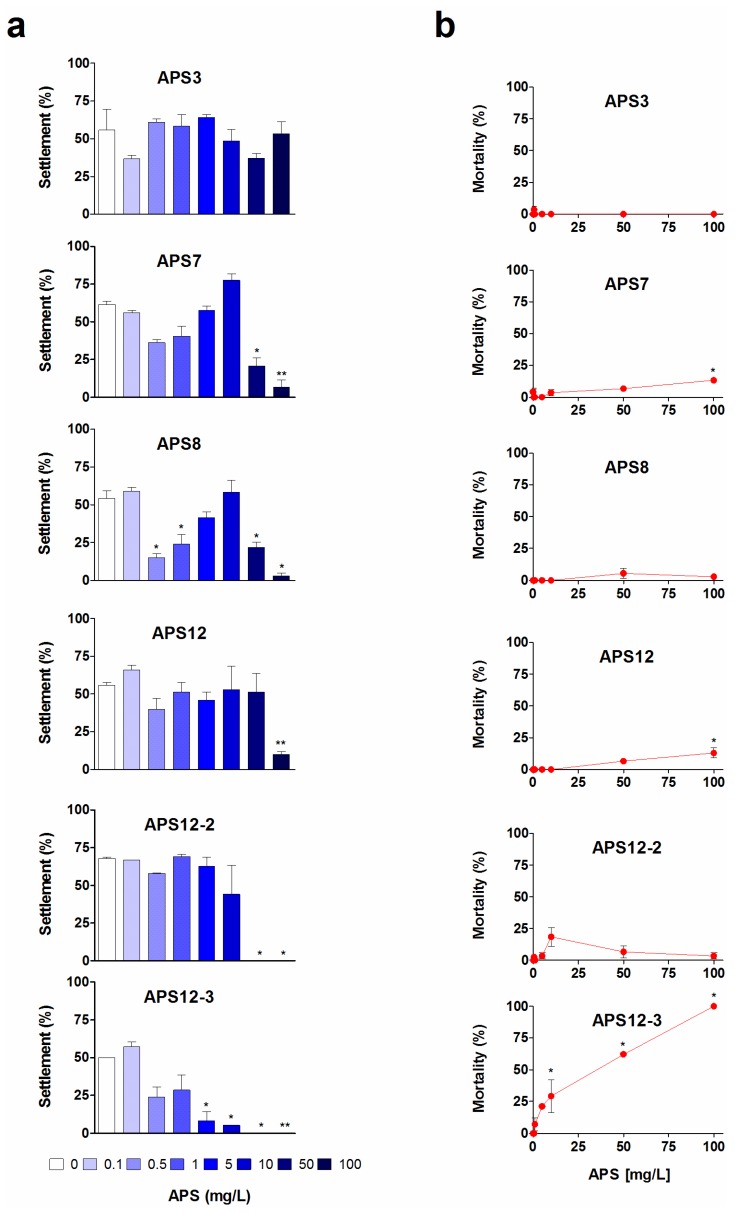
Anti-settlement activity (**a**) and toxicity (**b**) of synthetic poly-APS on *A. amphitrite* cyprids after 72-h exposure to the different poly-APS concentrations (as indicated). Data are expressed as the means ± standard error. * *p* < 0.05; ** *p* < 0.001.

We investigated the settlement of laboratory-reared cyprids of *A. amphitrite* after 24 h, 48 h and 72 h of exposure to these synthetic poly-APS in 24-well plates (see the Experimental Section). The results of the 72 h anti-settlement assays are shown in Figure 2a. 

APS8 was the most effective of the synthetic poly-APS for the inhibition of the settlement of cyprids (*p* < 0.01; Figure 2a). With an EC_50_ of 0.32 mg/L after 24 h (Table 1), APS8 matches the anti-settlement activity of natural poly-APS (EC_50_, 0.27 mg/L). Moreover, when these are expressed as molar concentrations, APS8 is almost two-fold more effective than natural poly-APS (Table 1). The biphasic dose response seen for APS8 (Figure 2a) is characterized by a significant inhibition of settlement at 0.5 and 1 mg/L, followed by the loss of anti-settlement activity at concentrations from 5 to 10 mg/L and then by the return of inhibition at 50 and 100 mg/L, thus suggesting the phenomenon of hormesis [44]. The underlying mechanism of such adaptive responses might be mediated via specific receptor and/or cell-signaling pathways [45,46]. For natural poly-APS, one of the possible molecular mechanisms of this anti-settlement activity is interference with the cyprid cholinergic system [28]. A recent novel hypothesis proposed that the cellular quality control systems that are involved in the recognition, repair and prevention of cell stress represent the underlying molecular mechanisms that account for the benefits of hormesis [47]. 

We observed a hormetic-like response also for APS7, even though this is not supported by *a posteriori* comparison of the means and less clearly for APS12. However, for both analogues, 50% inhibition of the cyprid settlement occurred at concentrations that are an order of magnitude higher than APS8 (Table 1). For all of these tested synthetic poly-APS, the settlement inhibition decreased with the time of exposure. This effect is particularly evident for polymer APS3, which shows no significant effect on the cyprid settlement (*p* = 0.45; F = 1.08) and is thus less interesting for further antifouling research.

**Figure 3:**
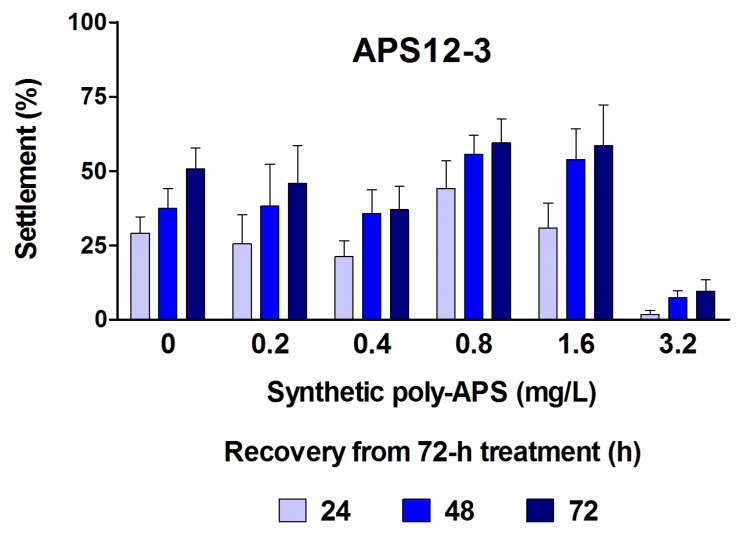
Recovery of *A. amphitrite* cyprid settlement in fresh filtered natural seawater after 72-h treatment with different concentrations of APS12-3 monitored for three consecutive days. Data are the means ± standard error.

The synthetic polymers, APS12-2 and APS12-3, inhibit the settlement of cyprids in a concentration-dependent manner, with the respective EC_50_ of 8.78 and 0.89 mg/L at 24 h (Table 1). As reported by Zovko *et al.* [40], APS12-3 has the highest antibacterial and antifungal activity among all of these synthetic poly-APS. By comparing the data for the inhibition of cyprid settlement with those for the toxicity of APS12-3 (Figure 2b), for the longer incubation times of 48 h and 72 h, it can be concluded that the inhibitory effects probably occur due to the toxic action of APS12-3. Interestingly, however, the potent inhibition of cyprid settlement by APS12-3 is reversible at concentrations up to 1.6 mg/L (Figure 3). The other synthetic poly-APS tested here did not show any significant toxic effects against these barnacle cyprids at concentrations of up to 100 mg/L (Figure 2b).

**Table 1:** Antifouling activities of natural poly-APS, Zn and Cu pyrithiones, and the synthetic poly-APS, assessed as the settlement (EC_50_) and mortality (LC_50_) of *A. amphitrite* cyprids.

Compound	Treatment (h)	EC_50_ (mg/L)	LC_50_ (mg/L)	EC_50_ (μM)	LC_50_ (μM)
Poly-APS ^1^	24	0.27 (0.15–0.47)		0.049	
Zn pyrithione ^1^	24	0.02		0.063	
Cu pyrithione ^1^	24	<0.01		<0.032	
APS3	24	5.72 (4.24–7.72)	>100	3.9	
	48	>100	>100		
	72	>100	>100		
APS7	24	10.50 (8.47–13.01)	>100	4.5	
	48	25.86 (23.29–28.71)	>100	11.1	
	72	29.38 (26.17–32.99)	>100	12.6	
APS8 ^2^	24	0.32 (0.26–0.39)	>100	0.026	
	48	0.50 (0.36–0.70)	>100	0.042	
	72	2.33 (1.78–3.04)	>100	0.195	
APS12	24	*nc*	>100		
	48	*nc*	>100		
	72	49.82 (37.18–66.76)	>100	4.0	
APS12-2	24	8.78 (8.37-9.20)	>100	0.597	
	48	9.38 (8.76–10.05)	>100	0.638	
	72	11.13 (10.38–11.94)	>100	0.757	
APS12-3 ^2^	24	0.89 (0.48–1.65)	61.13 (51.65–72.36)	0.146	10.0
	48	4.03 (3.49–4.65)	24.24 (20.09–29.24)	0.661	4.0
	72	4.76 (4.44–5.11)	17.97 (14.88–21.70)	0.781	2.9

Data are expressed as EC_50_ or LC_50_ (95% confidence interval); EC_50_, effective concentration that inhibits 50% of the cyprid population settlement; LC_50_, lethal concentration that kills 50% of the cyprid population; *nc*, not calculable; ^1^ data measured after a 24 h-treatment with poly-APS or commercial co-biocide [32]; ^2^ EC_50_ calculated from an additional experiment (see the Experimental Section).

### 2.2. Naupliar Mortality, Immobility and Swimming Speed Alteration Assays

**Figure 4:**
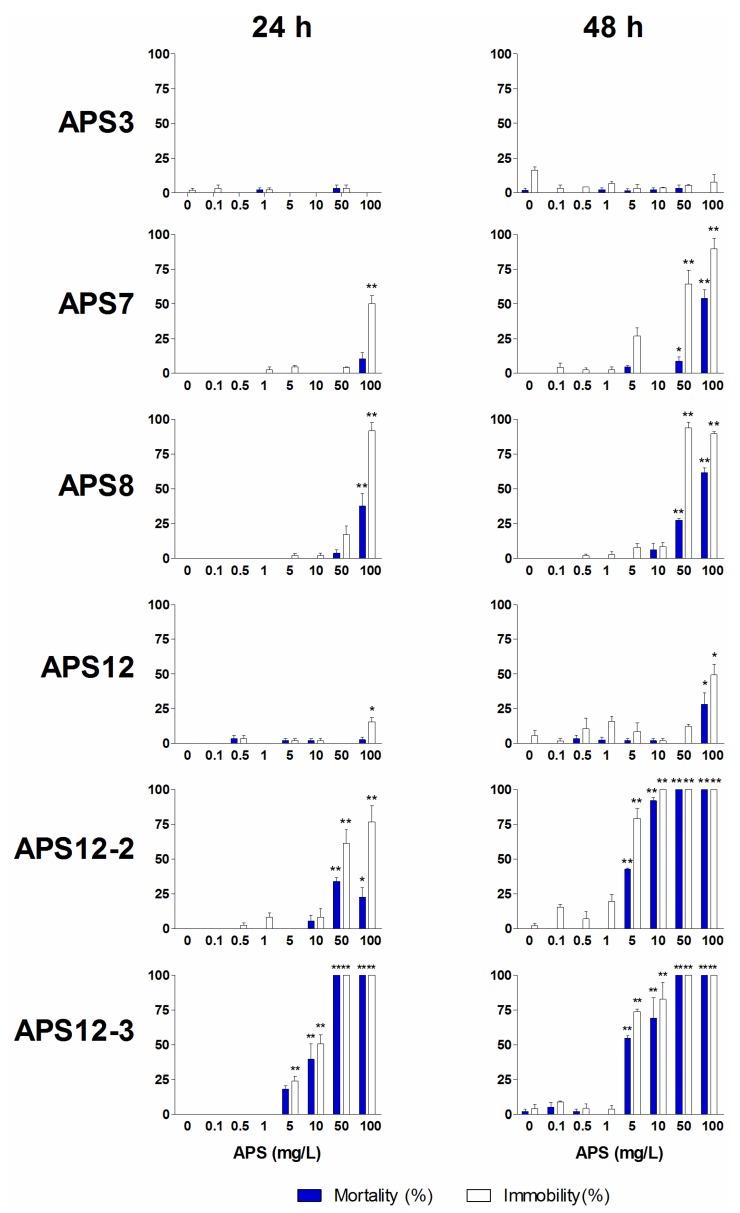
Immobility (white bars) and mortality (blue bars) of the synthetic poly-APS for *A. amphitrite* II stage nauplii after 24 h and 48 h exposure. Data are the means ± standard error. * *p* < 0.05; ** *p* < 0.001.

*Amphibalanus amphitrite* nauplii normally swim continuously, and their inability to move is evidence of toxicity [48]. On this basis, we measured the acute (mortality) and sub-acute (immobility) toxicity end-points of II stage nauplii exposed to the different solutions of the synthetic poly-APS for 24 h and 48 h. These immobility and naupliar mortality results are shown in Figure 4. 

**Figure 5:**
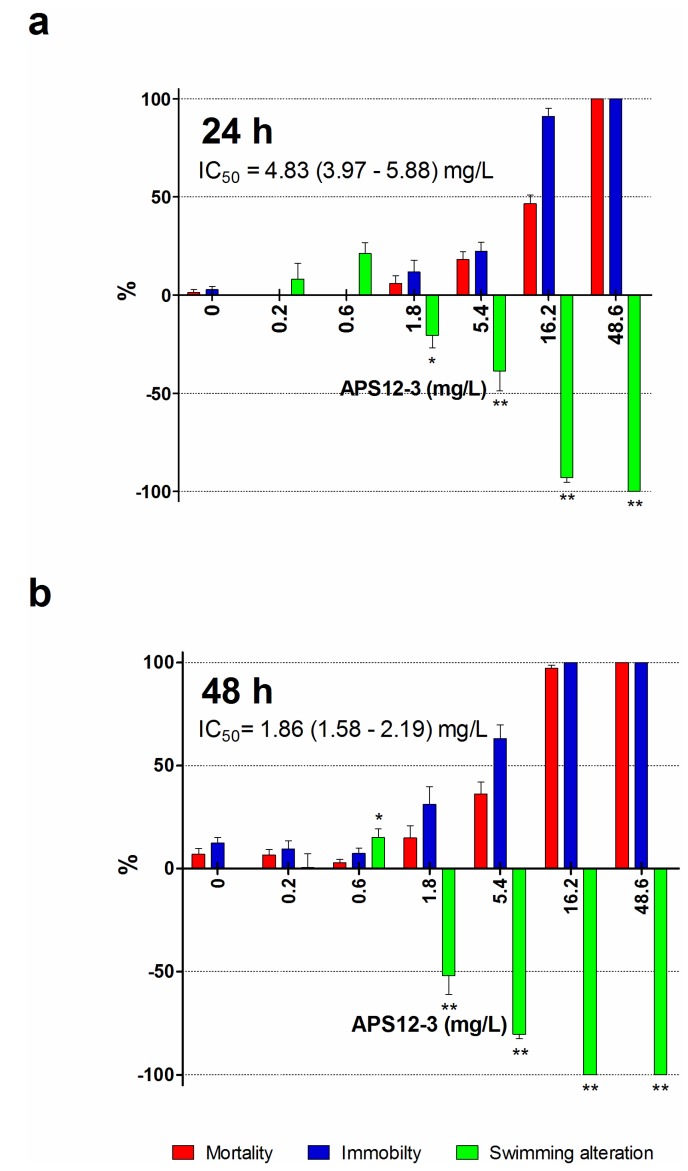
Swimming speed alteration (green bars), immobility (blue bars) and mortality (red bars) of the APS12-3 for *A. amphitrite* II stage nauplii after 24-h (**a**) and 48-h (**b**) incubations. Data are the means ± standard error. * *p* < 0.05; ** *p* < 0.001.

In ecotoxicological studies, the behavioral changes are more sensitive, short-term indicators of chemical toxicity than the assessment of lethal effects. Therefore, we investigated the sub-lethal toxicity of one of the synthetic analogues with the highest anti-settlement activity, APS12-3, using a swimming speed alteration test (Figure 5). Due to a lack of compound, the same evaluation has not been performed also for APS8. The synthetic poly-APS APS12-3 inhibited the mobility of nauplii in a concentration-dependent manner. The EC_50_ for immobility was 9.43 mg/L (after 24 h), and the ability of the nauplii to swim was completely lost at 50 mg/L APS12-3, and above. Thus, APS12-3 is moderately toxic to these nauplii, and indeed, the mortality (LC_50_ of 11.60 mg/L after 24 h) is three-fold greater compared to that for natural poly-APS (24-h LC_50_ of 30.01 mg/L; Table 2). The greater toxicity of APS12-3 towards this model organism, compared to natural poly-APS, is evidenced also by their 24-h swimming speed inhibition IC_50_ of 4.83 and >10 mg/L, respectively.

Increasing concentrations of APS12-2 also progressively inhibited the naupliar mobility and resulted in increased mortality. Prolonging the time of exposure to APS12-2 increased this immobility of nauplii (EC_50_, 36.92 mg/L at 24 h; 2.28 mg/L at 48 h). The toxicity of APS12-2 towards these nauplii is comparable to that of APS12-3.

**Table 2:** Lethal (mortality) and sub-lethal (immobility) toxicity of natural poly-APS, Zn and Cu pyrithiones and the synthetic poly-APS, as assessed with *A. amphitrite* II stage nauplii.

Compound	Treatment (h)	EC_50_ (mg/L)	LC_50_ (mg/L)	EC_50_ (μM)	LC_50_ (μM)
Poly-APS ^1^	24	>10	30.01 (21.71–41.49)	>1.81	5.43
Zn pyrithione ^1^	24	0.23 (0.16–0.33)	0.19 (0.13–0.30)	0.725	0.6
Cu pyrithione ^1^	24	0.03 (0.03–0.04)	<0.01	0.095	<0.032
APS3	24	>100	>100		
	48	>100	>100		
APS7	24	>100	>100		
	48	30.64 (25.43–36.9)	94.02 (81.73–108.17)	13.1	40.3
APS8	24	>100	>100		
	48	>100	79.37 (68.29–92.25)		6.7
APS12	24	>100	>100		
	48	>100	>100		
APS12-2	24	36.92 (29.33–46.46)	>100	2.5	
	48	2.28 (1.95–2.67)	4.80 (4.21–5.46)	0.15	0.32
APS12-3	24	9.43 (8.10–10.97)	11.60 (10.06–13.38)	1.5	1.9
	48	3.61 (3.14–4.16)	5.44 (4.64–6.37)	0.59	0.89

Data are expressed as EC_50_ or LC_50_ (95% confidence interval); EC_50_, effective concentration that inhibits mobility of 50% naupliar population; LC_50_, lethal concentration that kills 50% of the naupliar population; ^1^ data measured after a 24 h-treatment with poly-APS or commercial co-biocide [32].

At concentrations above 10 mg/L, APS7 and APS8 inhibited the naupliar mobility. This effect was more prominent after 48-h exposure at the higher doses of 50 mg/L and 100 mg/L APS7 and APS8. Both APS7 and APS8 showed a low toxicity towards these nauplii, with 48-h LC_50_ values of 94.02 mg/L and 79.37 mg/L, respectively.

The naupliar immobility was only minor upon exposure to APS3 and APS12.

A therapeutic ratio (TR; lethal concentration for 50% mortality (LC_50_) divided by the effective concentration for 50% inhibition of settlement (EC_50_)) is commonly used to evaluate compounds’ potential [2,26]. Generally, compounds with a TR > 50 and EC_50_ < 5 mg/L are considered to be promising non-toxic antifouling candidates [25]. We calculated TR values both for naupliar (TR_N_) and cyprids (TR_C_) toxicity (Table 3). TR_C_ denotes an antifouling mechanism (toxic or non-toxic), whereas TR_N_ indicates an environmental impact of a compound, since it is calculated by using LC_50_ towards non-target organisms (e.g., nauplii as representatives of the plankton) [49].

After a 24-h treatment of cyprids with synthetic analogue APS12-3, this analogue has a very promising antifouling activity (EC_50_ = 0.89 mg/L), and it seems to act through a non-toxic mechanism with a TR_C_ of 68.68. However, it is potentially hazardous for the environment (TR_N_ = 13.3). After 72 h, the antifouling activity of APS12-3 is less significant, and the compound displays a high toxicity towards target and non-target organisms (TR_C_ = 3.77 and TR_N_ = 1.35, respectively).

Another synthetic analogue, APS8, shows an anti-settlement activity (EC_50_, 0.32 mg/L) similar to natural poly-APS (EC_50_, 0.27 mg/L). Its TR_N_ is higher than that of the natural compound (158.74 and 111.15, respectively) suggesting an even lower environmental risk. Based on this, we propose its use as one of the pharmacophores in the prospective studies of non-toxic antifouling compounds.

**Table 3:** Therapeutic ratio values calculated using both data of naupliar (TR_N_) and cyprids (TR_C_) toxicity.

Compounds	Treatment (h)	EC_50_ (mg/L)	LC_50(N)_ (mg/L)	LC_50(C)_ (mg/L)	TR_C_	TR_N_
Poly-APS ^1^	24	0.27	30.01	/	/	111.15
Zn pyrithione ^1^	24	0.02	0.19	/	/	9.50
Cu pyrithione ^1^	24	<0.01	<0.01	/	/	/
APS3	24	5.72	>100	>100	*nc*	*nc*
	48	*nc*	>100	>100	*nc*	*nc*
	72	*nc*	*/*	>100	*nc*	*/*
APS7	24	10.50	>100	>100	*nc*	*nc*
	48	25.86	94.02	>100	*nc*	3.64
	72	29.38	/	>100	*nc*	/
APS8	24	0.32	>100	>100	*nc*	
	48	0.50	79.37	>100	*nc*	158.74
	72	2.33	/	>100	*nc*	/
APS12	24	*nc*	>100	>100	*nc*	*nc*
	48	*nc*	>100	>100	*nc*	*nc*
	72	49.82	/	>100	*nc*	/
APS12-2	24	8.78	>100	>100	*nc*	11.39
	48	9.38	4.8	>100	*nc*	0.51
	72	11.13	/	>100	*nc*	/
APS12-3	24	0.89	11.6	61.13	68.68	13.3
	48	4.03	5.44	24.24	6.01	1.35
	72	4.76	/	17.97	3.77	/

^1^ Data measured after a 24 h-treatment with poly-APS or commercial co-biocide [32]; *nc*, not calculable.

## 3. Experimental Section

### 3.1. Chemicals

Stock solutions (100 mg/L) of each synthetic poly-APS were prepared by dissolving them in filtered (0.22 μm) natural seawater. The information on the synthesis and NMR analyses of APS8, APS12 and APS12-2 is available in [39] and of APS3, APS7 and APS12-3 in [40]. The chemical structures of the synthetic poly-APS are illustrated in Figure 1. The Zn pyrithione (Zinc Omadine^®^) and Cu pyrithione (Copper Omadine^®^) were from Arch Chemicals Incorporated, Atlanta, USA.

### 3.2. Rearing of *Amphibalanus amphitrite* Larvae

Adult barnacles were maintained in aerated, filtered (0.45 μm) natural seawater at 20 °C, on a 16-h:8-h light-dark cycle. They were fed every two days with *Artemia salina* (50 to 100 mL; 200 larvae/mL) and *Tetraselmis suecica* (100 to 200 mL; 2 × 10^6^ cells/mL). The seawater was changed three times per week, and the barnacles were periodically rinsed with fresh water to remove epibionts or debris.

To obtain the nauplii for cyprid cultures, the adults were left to dry for 30 min to 40 min and then immersed in fresh seawater. The hatched nauplii were attracted to a light source and collected using a Pasteur pipette. They were reared to the cyprid stage as described in [50], by keeping them at 28 °C in natural filtered (0.22 μm) seawater and feeding them three times a week with *Tetraselmis suecica* (2 × 10^6^ cells/mL). In these conditions nauplii reach the cyprids stage in 5–6 days. The cyprids were harvested by filtration and aged for 4 days prior to use, in filtered (0.45 μm) natural seawater at 4 °C in the dark [48].

### 3.3. Settlement Assay

The effects of poly-APS on the barnacle cyprids settlement were tested using these *A. amphitrite* cyprids. In the preliminary settlement assays, all poly-APS-like compounds were tested within a wide range of concentrations (from 0 to 100 mg/L). The settlement assays were conducted in 24-well microplates (Greiner Bio-One International AG, Austria) by adding 20 to 25 cyprids per well, with each well containing 2 mL of the relevant poly-APS solution (*i.e*., 0.1, 0.5, 1, 5, 10, 50, 100 mg/L), or control seawater (*i.e*., 0 mg/L poly-APS). For each compound, the experiment was performed in duplicate (two wells per concentration).

The test plates were sealed to prevent evaporation and incubated at 28 °C in the dark. The settlement was evaluated after 24 h, 48 h and 72 h of incubation. The larvae were examined under a dissecting microscope, to record the number of dead and permanently attached and metamorphosed individuals. The experiments were terminated by the addition of three droplets of 40% formaldehyde into each test well and the counting of the settled and non-settled larvae. The results were expressed as the percentages (±standard error) of the settlement of the total number of larvae incubated (20–25). The EC_50_ was determined as the concentration of poly-APS causing 50% inhibition of the cyprids’ population settlement.

The settlement assay was repeated only for those synthetic poly-APS-like compounds that showed the best antifouling activities: APS8 and APS12-3. To better define the EC_50_, the assays were slightly modified in terms of the range of concentrations of these poly-APS solutions (0, 0.3, 0.6, 1.2, 2.4, 4.8, 9.6, 19.2 mg/L for APS8; 0, 0.2, 0.4, 0.8, 1.6, 3.2, 6.4, 12.8 mg/L for APS12-3), and the experiments were performed with three replicates (three wells per concentration). The data for the inhibition of cyprid settlement are not illustrated, but EC_50_ values are reported in Table 1.

As APS12-3 caused significant mortality of these cyprids at the higher concentrations, another experiment was designed to study the recovery of the settlement ability of these cyprid larvae. Briefly, after the 72 h treatment with APS12-3, the unsettled cyprids were collected from the wells, rinsed with filtered seawater and transferred into new microplates with clean fresh filtered (0.45 μm) natural seawater. The percentages of settled cyprids were then determined after 24 h, 48 h and 72 h at 28 °C.

### 3.4. Toxicity Assay

Acute (mortality) and sub-acute (immobility) toxicities of the synthetic poly-APS were assessed using II stage nauplii or cyprids of *A. amphitrite*. All of these tests were performed in duplicate (two wells per concentration) in 24-well microplates (Greiner Bio-One International AG, Kremsmünster, Austria), each well containing 2 mL of the relevant poly-APS solution (*i.e*., 0.1, 0.5, 1, 5, 10, 50 and 100 mg/L), or filtered seawater as control (*i.e.*, 0 mg/L poly-APS), with 20 to 25 larvae per well.

The naupliar mortality and immobility were evaluated after 24 h and 48 h of incubation at 20 °C in the dark, while the cyprids’ mortality was evaluated after 24 h, 48 h and 72 h of incubation at 28 °C. After the exposure to the relevant poly-APS or control filtered seawater, the larvae were examined under a dissecting microscope, and the number of dead larvae was recorded. The data are presented as the percentage of mortality ± standard error, and the LC_50_ is expressed as the concentration of the poly-APS that induced death in 50% of the tested organisms. When assessing naupliar immobility, the number of immobile larvae is expressed as the sum of dead larvae (not swimming and moving appendages for 10 s of observation) and non-swimming larvae (not shifting their barycenter, but moving their appendages). The EC_50_ was calculated as the concentration of the toxicant that caused 50% immobility of the exposed organisms after 24 and 48 h.

### 3.5. Naupliar Swimming Speed Test

A swimming speed assay [51,52] was used to measure the behavioral effects of the synthetic analogue APS12-3 on the *A. amphitrite* larvae. Briefly, II stage nauplii (15–20 per well) were exposed to the APS12-3 test solutions in 24-well microplates for 48 h, at 20 °C in the dark, without aeration and feeding. All of these experiments were performed in duplicate (two wells per concentration).

A Swimming Behavioral Recorder System (e-magine IT, Genova, Italy) was used to track the paths of the swimming nauplii. Prior to the video recording under infrared light, the nauplii were adapted to the dark for 2 min, to gain their steady swimming speeds and to reach a uniform spatial distribution. The swimming behavior was monitored in the dark at 20 °C, for about three seconds, at 25 frames/s. The resulting digital images were analyzed using advanced image processing software to reconstruct the individual naupliar swimming paths and to measure the average swimming speed (mm/s) for each of the nauplii (15–20 nauplii). Finally, the data are expressed as percentages in terms of the swimming alteration, after normalization to the mean swimming speed (*v*) of the control (filtered natural seawater), as follows (Equation 1):

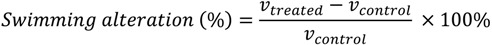
(1)


The IC_50_ value was determined as the concentration of APS12-3 that caused changes in swimming behavior in 50% of the test nauplii.

### 3.6. Statistical Analyses

The EC_50_ for the cyprid settlement inhibition after 24, 48 and 72 h, the EC_50_ for the naupliar immobility after 24 and 48 h, the LC_50_ for the larval mortality after 24, 48 and 72 h and the IC_50_ for naupliar swimming speed alteration were calculated using a trimmed Spearman–Karber analysis [53]. One-way analysis of variance (ANOVA) was performed, with the level of significance set at *p* < 0.05 or *p* < 0.001, followed by Student–Newman–Keuls (SNK) tests to compare the treatment means [54].

## 4. Conclusions

The present study was designed to determine the antifouling potential of six synthetic poly-APS that differ in the lengths of their alkyl chains (3–12 carbon atoms), their degree of polymerization (eight to 63 monomeric subunits) and the nature of their counter ions (chloride or bromide). Although several studies have reported that these structural features influence the biological activities of amphiphilic compounds, particularly in terms of their membrane-damaging potential and antimicrobial activities [55,56,57,58,59], we were not able to observe such a relationship.

The antifouling activity of natural poly-APS is believed to derive from its surfactant-like properties, or is potentially due to the inhibition of the cholinergic system, which is involved in cyprid settlement [28]. The underlying antifouling mechanism of these synthetic poly-APS remains unsolved; however, the hormetic responses observed imply the involvement of receptor system(s). Recently, a strong interaction between the synthetic poly-APS APS8 and human α7 nicotinic acetylcholine receptors was reported [60]. Acetylcholine serves as a neurotransmitter/neuromodulator during the settlement of *A. amphitrite* larvae [50], and therefore, the antifouling activity of APS8 and possibly also of these other synthetic poly-APS might derive from the binding of these synthetic poly-APS to these receptors and the subsequent competition with acetylcholine.

Although numerous natural products with anti-settlement activities have been reported to date, only a few have shown potential for commercialization [24]. Among these, natural poly-APS has been suggested as a non-toxic natural antifoulant [22,26]. With the recent chemical synthesis of various poly-APS [39,40] now ensuring an adequate supply of these compounds, we focused our current research on the finding of synthetic poly-APS with comparable or improved antifouling activities to natural poly-APS. Among the polymers tested, APS8 prevented the settlement of *A. amphitrite* cyprids via a non-toxic mechanism and with similar potency to natural poly-APS. On the other hand, APS12-3 showed an anti-settlement efficacy similar to the natural poly-APS, but with higher toxicity. 

## References

[1] Kirschner C.M., Brennan A.B. (2012). Bio-Inspired Antifouling Strategies. Annu. Rev. Mater. Res..

[2] Briand J.-F. (2009). Marine antifouling laboratory bioassays: An overview of their diversity. Biofouling.

[3] Jain A., Bhosle N.B. (2009). Biochemical composition of the marine conditioning film: Implications for bacterial adhesion. Biofouling.

[4] Dobretsov S., Dürr S., Thomason J.C. (2009). Marine Biofilms. Biofouling.

[5] Railkin A.I., Ganf T.A., Manylov O.G. (2003). Biofouling as a Process. Marine Biofouling: Colonization Processes and Defenses.

[6] Joint I., Tait K., Callow M.E., Callow J.A., Milton D., Williams P., Cámara M. (2002). Cell-to-cell communication across the prokaryote-eukaryote boundary. Science.

[7] Hadfield M.G., Paul V.J., McClintock J.B., Baker B.J. (2001). Natural chemical cues for settlement and metamorphosis of marine-invertebrate larvae. Marine Chemical Ecology.

[8] Hadfield M.G. (2011). Biofilms and marine invertebrate larvae: What bacteria produce that larvae use to choose settlement sites. Ann. Rev. Mar. Sci..

[9] Schultz M.P. (2007). Effects of coating roughness and biofouling on ship resistance and powering. Biofouling.

[10] Schultz M.P., Bendick J.A., Holm E.R., Hertel W.M. (2011). Economic impact of biofouling on a naval surface ship. Biofouling.

[11] Elimelech M., Phillip W.A. (2011). The future of seawater desalination: Energy, technology, and the environment. Science.

[12] Page H.M., Dugan J.E., Piltz F., Dürr S., Thomason J.C. (2009). Fouling and antifouling in oil and other offshore industries. Biofouling.

[13] Fitridge I., Dempster T., Guenther J., de Nys R. (2012). The impact and control of biofouling in marine aquaculture: A review. Biofouling.

[14] Dafforn K.A., Lewis J.A., Johnston E.L. (2011). Antifouling strategies: History and regulation, ecological impacts and mitigation. Mar. Pollut. Bull..

[15] Rosenhahn A., Schilp S., Kreuzer H.J., Grunze M. (2010). The role of “inert” surface chemistry in marine biofouling prevention. Phys. Chem. Chem. Phys..

[16] Sonak S., Pangam P., Giriyan A., Hawaldar K. (2009). Implications of the ban on organotins for protection of global coastal and marine ecology. J. Environ. Manag..

[17] Ralston E., Swain G. (2009). Bioinspiration—The solution for biofouling control?. Bioinspir. Biomim..

[18] Salta M., Wharton J.A., Stoodley P., Dennington S.P., Goodes L.R., Werwinski S., Mart U., Wood R.J.K., Stokes K.R. (2010). Designing biomimetic antifouling surfaces. Philos. Trans. A Math. Phys. Eng. Sci..

[19] Scardino A.J., de Nys R. (2011). Mini review: Biomimetic models and bioinspired surfaces for fouling control. Biofouling.

[20] Banerjee I., Pangule R.C., Kane R.S. (2011). Antifouling coatings: Recent developments in the design of surfaces that prevent fouling by proteins, bacteria, and marine organisms. Adv. Mater..

[21] Rittschof D. (2000). Natural product antifoulants: One perspective on the challenges related to coatings development. Biofouling.

[22] Fusetani N. (2004). Biofouling and antifouling. Nat. Prod. Rep..

[23] Turk T., Frangež R., Sepčić K. (2007). Mechanisms of toxicity of 3-alkylpyridinium polymers from marine sponge *Reneira sarai*. Mar. Drugs.

[24] Raveendran T.V., Limna Mol V.P. (2009). Natural product antifoulants. Curr. Sci..

[25] Qian P.-Y., Xu Y., Fusetani N. (2010). Natural products as antifouling compounds: Recent progress and future perspectives. Biofouling.

[26] Fusetani N. (2011). Antifouling marine natural products. Nat. Prod. Rep..

[27] Turk T., Sepčić K., Mancini I., Guella G. (2008). 3-Akylpyridinium and 3-alkylpyridine compounds from marine sponges, their synthesis, biological activities and potential use. Studies in Natural Products Chemistry.

[28] Sepčić K., Turk T., Fusetani N., Clare A. (2006). 3-Alkylpyridinium compounds as potential non-toxic antifouling agents. Antifouling Compounds SE-4.

[29] Sepčić K., Guella G., Mancini I., Pietra F., Serra M.D., Menestrina G., Tubbs K., Maček P., Turk T. (1997). Characterization of anticholinesterase-active 3-alkylpyridinium polymers from the marine sponge *Reniera sarai* in aqueous solutions. J. Nat. Prod..

[30] Grandič M., Sepčić K., Turk T., Juntes P., Frangež R. (2011). *In vivo* toxic and lethal cardiovascular effects of a synthetic polymeric 1,3-dodecylpyridinium salt in rodents. Toxicol. Appl. Pharmacol..

[31] Malovrh P., Sepčić K., Turk T., Maček P. (1999). Characterization of hemolytic activity of 3-alkylpyridinium polymers from the marine sponge *Reniera sarai*. Comp. Biochem. Physiol. Part C Pharmacol. Toxicol. Endocrinol..

[32] Faimali M., Sepčić K., Turk T., Geraci S. (2003). Non-toxic antifouling activity of polymeric 3-alkylpyridinium salts from the Mediterranean sponge *Reniera sarai* (Pulitzer-Finali). Biofouling.

[33] Garaventa F., Faimali M., Sepčić K., Geraci S. (2003). Laboratory analysis of antimicrofouling activity of Poly-APS extracted from *Reniera sarai* (Porifera: Demospongiae). Biol. Mar. Mediterr..

[34] Qian P.-Y., Chen L., Xu Y. (2013). Mini-review: Molecular mechanisms of antifouling compounds. Biofouling.

[35] Mancini I., Defant A., Guella G. (2007). Recent synthesis of marine natural products with antibacterial activities. Anti-Infect. Agents Med. Chem..

[36] Mancini I., Sicurelli A., Guella G., Turk T., Maček P., Sepčić K. (2004). Synthesis and bioactivity of linear oligomers related to polymeric alkylpyridinium metabolites from the Mediterranean sponge Reniera sarai. Org. Biomol. Chem..

[37] Faimali M., Garaventa F., Mancini I., Sicurelli A., Guella G., Piazza V., Greco G. (2005). Antisettlement activity of synthetic analogues of polymeric 3-alkylpyridinium salts isolated from the sponge Reniera sarai. Biofouling.

[38] Chelossi E., Mancini I., Sepčić K., Turk T., Faimali M. (2006). Comparative antibacterial activity of polymeric 3-alkylpyridinium salts isolated from the Mediterranean sponge Reniera sarai and their synthetic analogues. Biomol. Eng..

[39] Houssen W.E., Lu Z., Edrada-Ebel R., Chatzi C., Tucker S.J., Sepčić K., Turk T., Zovko A., Shen S., Mancini I. (2010). Chemical synthesis and biological activities of 3-alkyl pyridinium polymeric analogues of marine toxins. J. Chem. Biol..

[40] Zovko A., Vaukner Gabrič M., Sepčić K., Pohleven F., Jaklič D., Gunde-Cimerman N., Lu Z., Edrada-Ebel R., Houssen W.E., Mancini I. (2012). Antifungal and antibacterial activity of 3-alkylpyridinium polymeric analogs of marine toxins. Int. Biodeterior. Biodegrad..

[41] Grandič M., Aráoz R., Molgó J., Turk T., Sepčić K., Benoit E., Frangež R. (2013). Toxicity of the synthetic polymeric 3-alkylpyridinium salt (APS3) is due to specific block of nicotinic acetylcholine receptors. Toxicology.

[42] Eleršek T., Kosi G., Turk T., Pohleven F., Sepčić K. (2008). Influence of polymeric 3-alkylpyridinium salts from the marine sponge Reniera sarai on the growth of algae and wood decay fungi. Biofouling.

[43] Holm E.R. (2012). Barnacles and biofouling. Integr. Comp. Biol..

[44] Calabrese E.J. (2013). Biphasic dose responses in biology, toxicology and medicine: Accounting for their generalizability and quantitative features. Environ. Pollut..

[45] Calabrese E.J., Mattson M.P. (2011). Hormesis provides a generalized quantitative estimate of biological plasticity. J. Cell Commun. Signal..

[46] Calabrese E.J. (2013). Hormetic mechanisms. Crit. Rev. Toxicol..

[47] Wiegant F.A.C., de Poot S.A.H., Boers-Trilles V.E., Schreij A.M.A. (2012). Hormesis and cellular quality control: A possible explanation for the molecular mechanisms that underlie the benefits of mild stress. Dose-Response.

[48] Rittschof D., Clare A.S., Gerhart D.J., Mary S.A., Bonaventura J. (1992). Barnacle *in vitro* assays for biologically active substances: Toxicity and settlement inhibition assays using mass cultured *Balanus amphitrite amphitrite* Darwin. Biofouling.

[49] Piazza V., Roussis V., Garaventa F., Greco G., Smyrniotopoulos V., Vagias C., Faimali M. (2011). Terpenes from the red alga *Sphaerococcus coronopifolius* inhibit the settlement of barnacles. Mar. Biotechnol..

[50] Faimali M., Falugi C., Gallus L., Piazza V., Tagliafierro G. (2003). Involvement of acetyl choline in settlement of *Balanus amphitrite*. Biofouling.

[51] Faimali M., Garaventa F., Piazza V., Greco G., Corrà C., Magillo F., Pittore M., Giacco E., Gallus L., Falugi C. (2006). Swimming speed alteration of larvae of Balanus amphitrite as a behavioural end-point for laboratory toxicological bioassays. Mar. Biol..

[52] Garaventa F., Gambardella C., di Fino A., Pittore M., Faimali M. (2010). Swimming speed alteration of *Artemia* sp. and *Brachionus plicatilis* as a sub-lethal behavioural end-point for ecotoxicological surveys. Ecotoxicology.

[53] Hamilton M.A., Russo R.C., Thurston R.V. (1977). Trimmed Spearman-Karber method for estimating median lethal concentrations in toxicity bioassays. Environ. Sci. Technol..

[54] De Muth J.E. (2006). Basic Statistics and Pharmaceutical Statistical Applications.

[55] Kleszczyńska H., Bielecki K., Sarapuk J., Bonarska-Kujawa D., Pruchnik H., Trela Z., Łuczyński J. (2009). Biological activity of new *N*-oxides of tertiary amines. Z. Naturforsch. C J. Biosci..

[56] Zarif L., Riess J.G., Pucci B., Pavia A.A. (1993). Biocompatibility of alkyl and perfluoroalkyl telomeric surfactants derived from THAM. Biomater. Artif. Cells Immobil. Biotechnol..

[57] Kuroda K., DeGrado W.F. (2005). Amphiphilic polymethacrylate derivatives as antimicrobial agents. J. Am. Chem. Soc..

[58] Sarapuk J., Kleszczyńska H., Pernak J., Kalewska J., Rózycka-Roszak B. (1999). Influence of counterions on the interaction of pyridinium salts with model membranes. Z. Naturforsch. C.

[59] Kleszczynska H., Sarapuk J., Rozycka-Roszak B. (1998). The role of counterions in the interaction of some cationic surfactants with model membranes. Pol. J. Environ. Stud..

[60] Zovko A., Viktorsson K., Lewensohn R., Kološa K., Filipič M., Xing H., Kem W.R., Paleari L., Turk T. (2013). APS8, a polymeric alkylpyridinium salt blocks α7 nAChR and induces apoptosis in non-small cell lung carcinoma. Mar. Drugs.

